# Overseas Nursing Association

**Published:** 1919-07-05

**Authors:** 


### OVERSEAS NURSING ASSOCIATION.

The activities of the Overseas Nursing Association, which have been necessarily curtailed by the war, have recovered with surprising rapidity, and a clear call to further effort in a wider field was given at the Association's Annual Meeting, held last Wednesday in Chelsea House, Cadogan Square, the residence of Sir Owen and Lady Phillips.

The most important development of the past year has been in Western Canada, where a large number of nurses have been sent in response to a growing need for maternity and other nurses. Recent events are accentuating those needs. It is estimated that over 25,000 British women have married Canadian soldiers and sailors, and that Canadian soldiers are marrying in this country at the rate of 1,000 a month. Their wives are now sailing for their new homes, and according to Col. J. Obed Smith, the Canadian authorities estimate that at least 40 per cent. of them are expectant mothers. In the conditions they will have to face in the Western Provinces they run very grave dangers in child-birth. The Women Grain Growers' Association of Saskatchewan stated that in 1914 "one woman in every three was materially injured in child-birth in the Province of Saskatchewan alone," and that infant mortality was twice as high as it need be. Women with a knowledge of midwifery were urgently needed, said Col. Smith.

General nurses, too, were greatly in demand, as the doctors in outlying towns were at present forced to employ anyone with a knowledge of nursing, however slight. "If you cannot send us a hundred trained nurses," he said, "let us have five hundred capable middle-aged women who can aid the already overworked nurses of Canada."

A plea for India was put forward by Sir Walter Egerton. Our only hospitals were on the coast, he said, and at certain inland stations, and there must be a great need of nurses in the Dependency. People must be brought to realise the importance of trained white nurses in outlying stations, not only for caring for the patients but for training coloured nurses to assist them. Insufficient Governmental aid was, however, forthcoming, and he suggested that in the interests of the Empire as a whole each Colonial Government should put by a small sum for the aid of white nurses within it.

Col. L. C. S. Amery, M.P., Under-Secretary for the Colonies, expressed the thanks of his Department for the work the Nursing Association was doing, and drew attention to their small expenditure, which he described as "totally disproportionate to the result." The need for nurses abroad, he continued, was growing; this might be termed the Age of Health, for never before had the problem of health and the prevention of the wastage of life been so prominent. Men and women were leaving for tropical countries in large numbers, ignorant of the conditions with which they would be faced. In the tropics the man was practically divorced from his family, who had to be left at home. In the interests of the country every measure that could be taken must be in order to remove these bad conditions, and allow a normal family life all over the world.

The question of the salaries of nurses abroad was raised by the Hon. Gideon Murray, M.P., who suggested that the Government should take a census of salaries with a view to their improvement. He cited the case of a matron in Santa Lucia, who for £175 a year, from which she had to pay for food and clothing, was expected to be in charge of a hospital of sixty patients, and had written during a recent pneumonia outbreak that she "had not been to bed for a fortnight." In St. Vincent a matron was receiving only £130 a year, and in both these cases much extra work had been imposed by the war, with no corresponding increase in assistance.

Viscount Gladstone, who occupied the chair, referred to the King Edward Order of Nurses, which during the war had splendidly held their own, but had not been able to add to their strength, but he prophesied that now they would continue to make progress. The demand for nurses all over the world could not be met; and after its period of comparative inactivity, during which it had been able to take stock of its own position, the Association was busily making up its arrears, and letting loose the pent-up energy of the last few years. There was a wider and still wider scope for its activities, and he urged the meeting not to rest satisfied with small or partial results.

Continuing her interest in the work of the Association, H.R.H. the Princess Beatrice was present, and a hearty vote of thanks to her, proposed by Col. Amery, was passed at the close of the meeting.

